# iATC-mHyb: a hybrid multi-label classifier for predicting the classification of anatomical therapeutic chemicals

**DOI:** 10.18632/oncotarget.17028

**Published:** 2017-04-11

**Authors:** Xiang Cheng, Shu-Guang Zhao, Xuan Xiao, Kuo-Chen Chou

**Affiliations:** ^1^ College of Information Science and Technology, Donghua University, Shanghai 201620, China; ^2^ Computer Department, Jingdezhen Ceramic Institute, Jingdezhen 333001, China; ^3^ Gordon Life Science Institute, Boston, MA 02478, USA; ^4^ Center for Informational Biology, University of Electronic Science and Technology of China, Chengdu 610054, China; ^5^ Center of Excellence in Genomic Medicine Research (CEGMR), King Abdulaziz University, Jeddah 21589, Saudi Arabia

**Keywords:** ATC classification, drug ontology, multi-label system, Chou's five intuitive metrics

## Abstract

Recommended by the World Health Organization (WHO), drug compounds have been classified into 14 main ATC (Anatomical Therapeutic Chemical) classes according to their therapeutic and chemical characteristics. Given an uncharacterized compound, can we develop a computational method to fast identify which ATC class or classes it belongs to? The information thus obtained will timely help adjusting our focus and selection, significantly speeding up the drug development process. But this problem is by no means an easy one since some drug compounds may belong to two or more than two ATC classes. To address this problem, using the DO (Drug Ontology) approach based on the ChEBI (Chemical Entities of Biological Interest) database, we developed a predictor called iATC-mDO. Subsequently, hybridizing it with an existing drug ATC classifier, we constructed a predictor called iATC-mHyb. It has been demonstrated by the rigorous cross-validation and from five different measuring angles that iATC-mHyb is remarkably superior to the best existing predictor in identifying the ATC classes for drug compounds. To convenience most experimental scientists, a user-friendly web-server for iATC-mHyd has been established at http://www.jci-bioinfo.cn/iATC-mHyb, by which users can easily get their desired results without the need to go through the complicated mathematical equations involved.

## INTRODUCTION

Based on their therapeutic and chemical characteristics, drug compounds are classified into 14 main categories, or 14 main ATC (Anatomical Therapeutic Chemical) classes (see, e.g., http://www.whocc.no/atc/structure_and_principles/).

Given an uncharacterized compound, can we develop a computational method to identify which ATC class it belongs to? The information thus obtained will timely help adjusting our focus and selection, significantly speed up the drug development process.

In a pioneer work, Dunkel et al. [1] proposed a computational method to identify the ATC classes of drug compounds based on their structural fingerprint information. Three years later, Chen et al. [2] developed an improved method by using the information of chemical-chemical interactions and chemical-chemical similarities. Actually, the ATC classification is a multi-label system [3], in which a same drug compound may belong to two or more different classes. To effectively deal with the difficulty caused by the multi-label nature, recently Cheng et al. [4] proposed a powerful predictor called “iATC-mISF” by incorporating the informations of the chemical-chemical interaction, structural similarity, and fingerprintal similarity into the sample formulation.

As is known, mapping the protein samples into the GO (gene ontology) database space [5–11] could significantly enhance the quality of predicting protein subcellular localization. Particularly when the proteins investigated might belong to two or more subcellular locations, as demonstrated by many publications for various different organisms [12–23], where the PseAAC (pseudo amino acid composition) approach [24, 25] was also adopted as a backup. Inspired by the successes of gene ontology approach, Chen et al. [26] proposed a drug ontology method to predict the ATC classification. The corresponding improvement, however, was not as remarkable as in the case of protein subcellular location prediction.

The present study was initiated in an attempt to propose a new DO (Drug Ontology) method for predicting the ATC classes of drug compounds by being based on the ontology via the ChEBI (Chemical Entities of Biological Interest) database [27].

## RESULTS AND DISCUSSION

A new predictor called iATC-mHyb has been established by hybridizing the iATC-mISF method [4] with the powerful iATC-mDO sub-predictor. The later is a newly constructed predictor with the DO approach via the ChEBI database. The reason to adopt such hybrid method is because (1) some drug compounds are not included in the current ChEBI database, and hence iATC-mDO cannot cover them although it is extremely powerful to those within the ChEBI database, and (2) the iATC-mISF had been the most powerful one among the existing ATC predictors [4].

Listed in Table 1 are the tested results by the new predictor iATC-mHyb on the benchmark dataset (see the section of MATERIALS AND METHODS later) via the most rigorous cross-validation method, the jackknife test [28, 29]. For facilitating comparison, listed in that table are also the corresponding results obtained by the iATC-mISF, the best one among the existing predictors for ATC classification. It can be seen from Table 1 that (1) the success rates obtained by the new predictor are all higher than those by iATC-mISF in “absolute true”, “accuracy”, “aiming”, and “coverage”, and that (2) the “absolute false” rate for the new predictor is almost 50% lower than that of the existing best predictor. As pointed out in a comprehensive review paper [3], among the aforementioned five metrics for the multi-label systems, the most important are “absolute true” and “absolute false”. It is extremely difficult to increase the absolute true rate and reduce the absolute false rate of a predictor for multi-label systems. Therefore, in reporting the results of their various prediction methods for multi-label systems, many investigators (see, e.g., [2, 12–17, 30–32] even did not mention the “absolute true” and “absolute false” rates. Actually, as pointed out by two recent papers [4, 33], the absolute true rates reported by most multi-label predictors (see, e.g. [23, 34]) were under 50%. In contrast to that, the 66.75% of absolute true achieved by the new predictor (Table 1) should be deemed a significantly improvement. Also, to our best knowledge, iATC-mHyb is the first multi-label predictor ever developed in biomedicine that can achieve lower than 3% of absolute false rate.

**Table 1:** The jackknife success rates achieved by iATC-mHyb and ATC-mISF on the benchmark dataset S of Eq.1 (cf. Supporting Information S1)

Predictor	Five metrics for multi-label system^a^				
	Aiming↑^b^	Coverage↑^b^	Accuracy↑^b^	Absolute true↑^b^	Absolute false↓^c^
iATC-mISF^d^	67.83%	67.10%	66.41%	60.98%	5.85%
iATC-mHyb^e^	71.91%	71.46%	71.32%	66.75%	2.43%

^a^See Eq.12 for the definitions of the five metrics used to measure the prediction quality for multi-label systems [3].

^b^The upper arrow means that the larger the rate the better the prediction quality is.

^c^The down arrow means that the smaller the rate the better the prediction quality is.

^d^The predictor proposed in [4].

^e^The predictor proposed in the current paper.

The aforementioned facts have indicated that, significant improvement can be achieved as well by adopting the DO approach.

Moreover, with its development, the ChEBI database will cover more and more drug compounds, and the iATC-mDO will further enhance its power, and so will the iATC-mHyb predictor.

As pointed out in [35], the publicly accessible web-servers represent the new direction and trend for developing new predictors or computational tools [33, 36–58]. Actually, papers with a user-friendly and publicly accessible web-server will significantly enhance their impacts [59]. In view of this, the web-server for iATC-mHyb has been established at http://www.jci-bioinfo.cn/iATC-mHyb.

To maximize users’ convenience, a step-to-step guide of how to use the iATC-mHyb web-server is given below.

**Figure 1:**
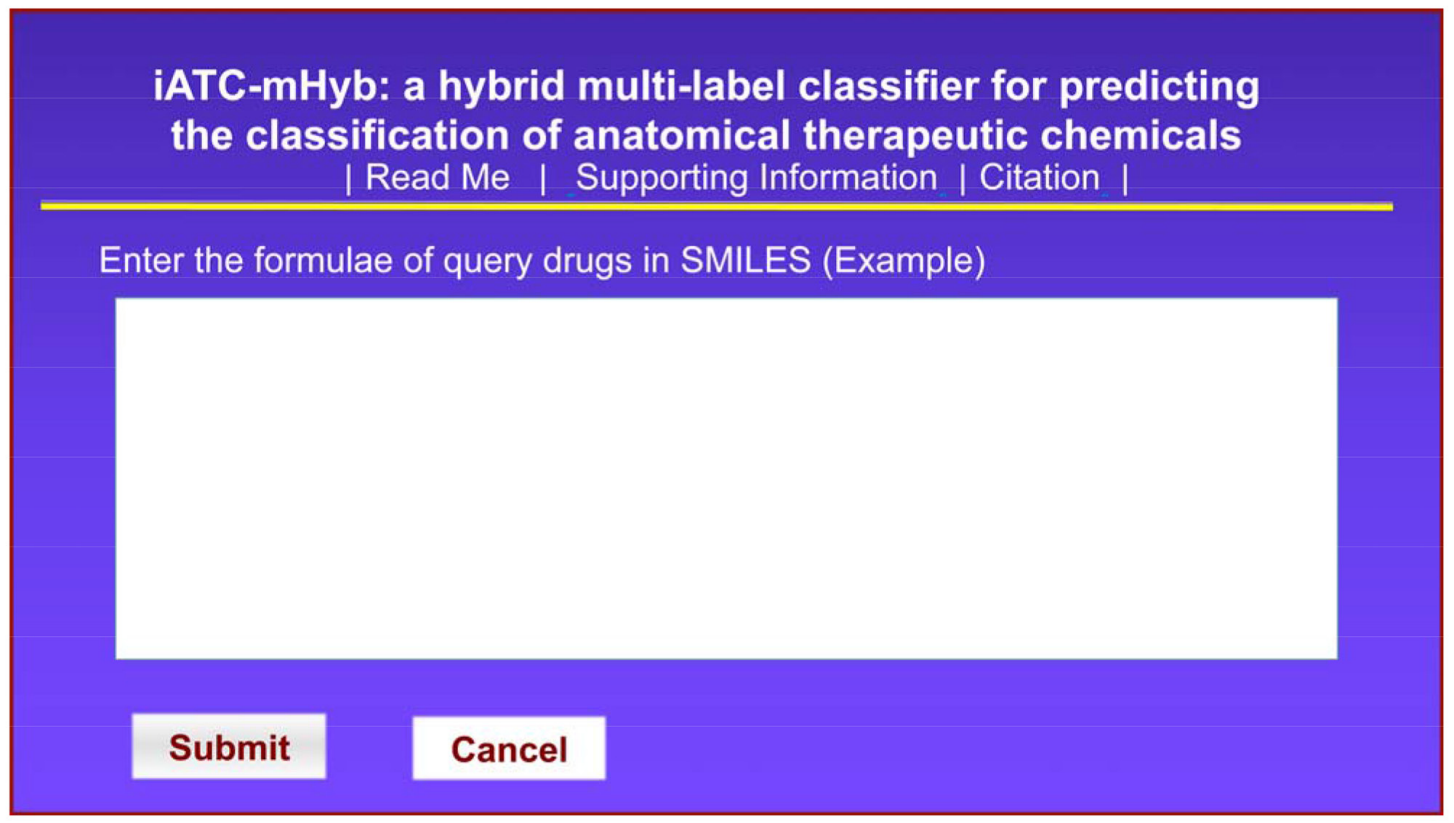
The semi-screenshot for the top page of the iATC-mHyb web-server, which is located at http://www.jci-bioinfo.cn/iATC-mHyb

**Step 1**. Open the web-server at http://www.jci-bioinfo.cn/iATC-mHyb, the top page of iATC-mHyb will appear on the computer screen, as shown in Figure 1. Click on the Read Me button to see a brief introduction about the iATC-mHyb and the caveat when using it.

**Step 2**. Either type or copy/paste the formulae of query compounds into the input box at the center of Figure 1. The input compounds should be in the SMILES format. For the example of compounds in SMILES format, click the Example button right above the input box.

**Figure 2:**
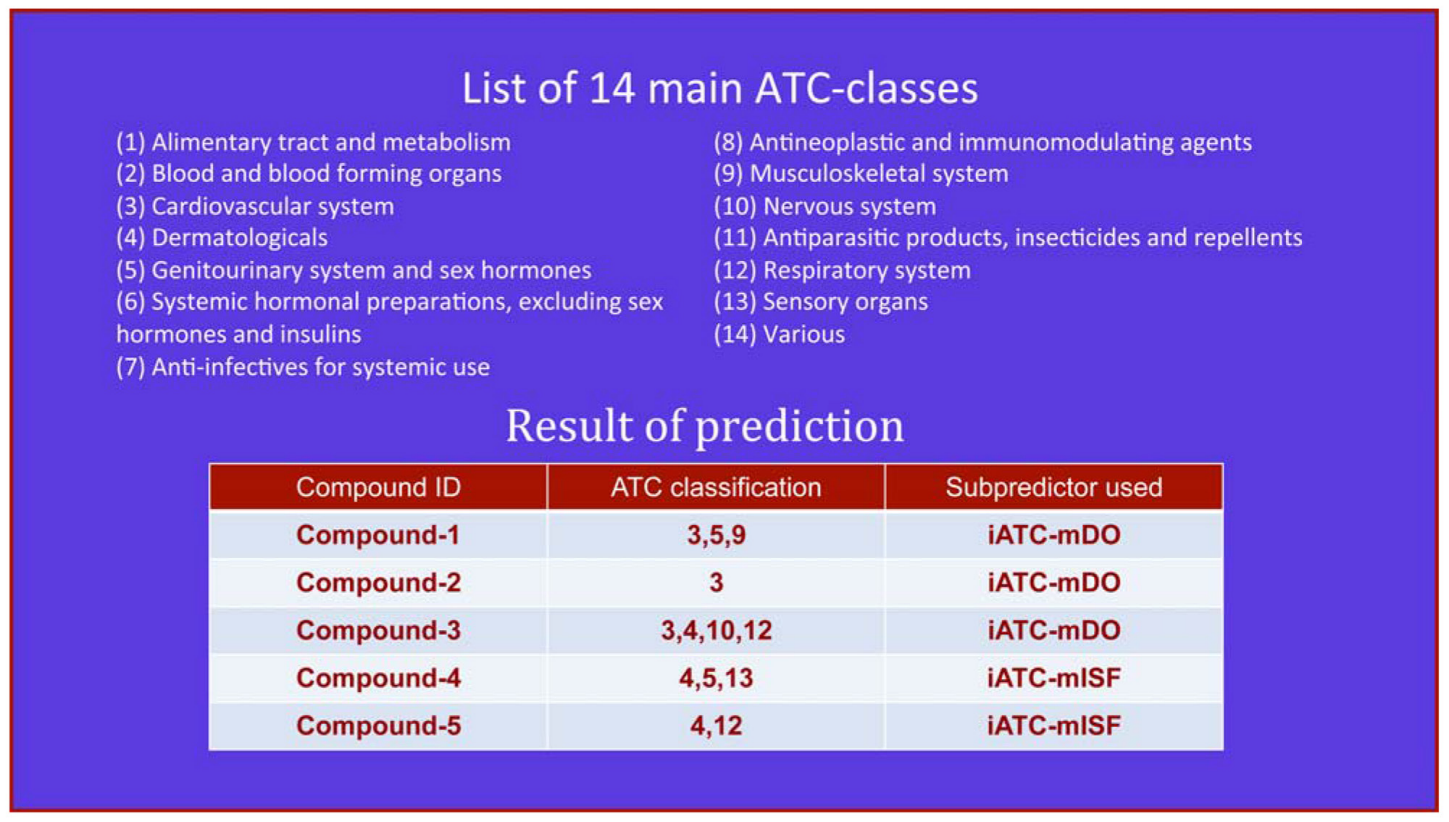
The semi-screenshot for the output generated by the Step 3 of users’ guide in the Results and Discussion section

**Step 3**. Click on the Submit button to see the predicted result. For example, if using the formulae of the five compounds in the Example window as the input, one will see Figure 2 shown on the computer screen, indicating the following results. (**1**) Compound-1 belongs to three different ATC-classes; i.e., classes 3, 5 and 9, which are predicted by iATC-mDO subpredictor, meaning that the compound is covered by the ChEBI database. (**2**) Compound-2 belongs to only one ATC-class; i.e., class 3, which is predicted by iATC-mDO subpredictor, meaning the compound is covered by the ChEBI database. (**3**) Compound-3 belongs to four different ATC-classes; i.e., classes 3, 4, 10 and 12, which are predicted by iATC-mDO subpredictor, meaning that the compound is covered by the ChEBI database. (**4**) Compound-4 belongs to three different ATC-classes; i.e., classes 4, 5 and 13, which are predicted by iATC-mISF subpredictor, meaning that the compound is not covered by the ChEBI database. (**5**) Compound-5 belongs to two different ATC-classes; i.e., classes 4 and 12, which are predicted by iATC-mISF subpredictor, meaning that the compound is also not covered by the ChEBI database. All these results are fully consistent with the experimental observations.

**Step 4**. Click on the Citation button to find the key relevant papers that have been used to document the detailed development and algorithm of iATC-mHyb.

**Step 5**. Click the Supporting Information button to download the all the “Supporting Information” files mentioned in this paper.

## MATERIALS AND METHODS

As demonstrated in a series of recent method-developing studies [33, 45–49, 51–55, 57, 60–65], to establish a really useful statistical predictor for a drug system, according to the Chou's 5-step rule [66] we should make the following five steps very clear: (**1**) how to construct or select a valid benchmark dataset to train and test the predictor; (**2**) how to formulate the drug compound samples with an effective mathematical expression that can truly reflect their essential correlation with the target concerned; (**3**) how to introduce or develop a powerful algorithm (or engine) to run the prediction; (**4**) how to properly conduct cross-validation tests to objectively evaluate the anticipated accuracy; (**5**) how to provide a web-server and user guide to make users very easily to get their desired results. Below, let us to address these point-by-point.

### Benchmark dataset

For facilitating comparison, in this study we used the same benchmark dataset (Supporting Information S1) as used in [2, 4]. It contains 3,883 drugs classified into the 14 main ATC-classes whose names in medicinal chemistry are given in Table 2. Thus, the benchmark dataset S can be formulated as

**Table 2:** Breakdown of the 3,883 drug compounds in the benchmark dataset S according to the 14 ATC classes (cf. Eq.1)

Subset	Name	Number of drugs
$S_{1}$	Alimentary tract and metabolism	540
$S_{2}$	Blood and blood forming organs	133
$S_{3}$	Cardiovascular system	591
$S_{4}$	Dermatologicals	421
$S_{5}$	Genito-urinary system and sex hormones	248
$S_{6}$	Systemic hormonal preparations, excluding sex hormones and insulins	126
$S_{7}$	Antiinfectives for systemic use	521
$S_{8}$	Antineoplastic and immunomodulating agents	232
$S_{9}$	Musculo-skeletal system	208
$S_{10}$	Nervous system	737
$S_{11}$	Antiparasitic products, insecticides and repellents	127
$S_{12}$	Respiratory system	427
$S_{13}$	Sensory organs	390
$S_{14}$	Various	211
Number of total virtual drugs		4,912^a^
Number of total structural different drugs		3,883^b^

^a^ The number of virtual drugs is counted as follows: for a structurally same drug, its contribution to the total number of virtual drugs is 2 if it occurs in two different ATC classes; that is 3 if it occurs in three different ATC classes; and so forth.

^b^ Of the 3,883 structural different drugs, 3,295 belong to one class, 370 to two classes, 110 to three classes, 37 to four classes, 27 to five classes, and 44 to six classes. See Supporting Information S1 for the detailed drug codes listed in each of 14 ATC-classes.

$$S=S_{1}\cup S_{2}\cup \cdots \cup S_{m}\cup \cdots \cup S_{13}\cup S_{14}$$ (1)

where the subset $S_{m}$ only contains the samples from the *m*-th ATC class (*m* = 1,2,3,...,14), and ∪ denotes the symbol for “union” in the set theory. Listed in Table 2 is a breakdown of the benchmark dataset according to the 14 subsets in Eq.1.

As we can see from the table, among the 3,883 drugs, 3,295 occur in one class, 370 in two classes, 110 in three classes, 37 in four classes, 27 in five classes, 44 in six classes, and none occurs in more than six classes. For such a multi-label system, let us use a more intuitive method to describe the benchmark dataset as given in Supporting Information S2, where the symbol “1” under the title of “ATC classification” means the drug concerned occurs in the corresponding class, “0” means not.

### Sample formulation

One of the keys in developing a powerful predictor is to formulate the samples with an effective mathematical expression that can truly reflect their intrinsic correlation with the target to be predicted [66]. In the previous paper [4], three different maximum score approaches were used to formulate the samples; they are (1) the interaction among the drug compounds concerned, (2) their structural similarity, and (3) their fingerprint similarity. Here, we are to address this problem by considering the maximum score in the DO (drug ontology) similarity; i.e., a sample in the benchmark dataset S of **Eq.1** is defined by

$$D^{\text{DO-Sim}}={\left[\begin{array}{ccc} \alpha_{1}\;\;\;\;\;\alpha_{2}\;\;\;\;\;\alpha_{3} & \cdots & \alpha_{14} \end{array}\right]}^{T}$$ (2)

where **T** is the transposition operator, α_1_ stands for its maximum DO similarity score with the drugs in the subset $S_{1}$, α_2_ for its maximum DO similarity score with the drugs in the subset $S_{2}$, α_3_ for that in subset $S_{3}$, and so forth. These DO similarity scores can be easily calculated [67, 68] from the ChEBI database [27] via KEGG [69].

Note that, of the 3,833 drug compounds in the benchmark dataset, only 1,144 can be found in the current ChEBI database (ftp://ftp.ebi.ac.uk/pub/databases/chebi/ontology/), and can be defined by Eq.2. For remaining (3,883 – 1,144) = 2,689 samples that are not included in the ChEBI, they will be expressed by the formulation in [4] and treated by the method described there. For clarity, let us use $S^{\text{DO}}\subset S$ to denote the 1,144 samples that occur in the current ChEBI database. The 1,144 drug compounds in the subset $S^{\text{DO}}$ are given in the Supporting Information S3.

### Operation algorithm

In this study, the ML-GKR (multi-label Gaussian kernel regression) classifier has been adopted to predict the ATC-classes, as described below.

Suppose the *i*-th drug in the benchmark dataset $S^{\text{DO}}$ can be formulated as

$$D^{i}=\;{\left[\alpha_{1}^{i}{}_{\;\;\;}\;\alpha_{2}^{i}\;\;\;\;\alpha_{3}^{i}\;\;\;\;\cdots \;\;\;\;\alpha_{14}^{i}\right]}^{T}(i=1,2,\;\cdots ,\;1144)$$ (3)

And its attribution in a multi-label system can be formulated as a vector **L***^i^* given by

$$L^{i}={\left[\begin{array}{ccc} \ell_{1}^{i}\;\;\;\;\;\ell_{2}^{i}\;\;\;\;\ell_{3}^{i} & \cdots & \ell_{14}^{i} \end{array}\right]}^{T}$$ (4)

where

$$\ell_{m}^{i}=\{\begin{array}{ll} +1 & \text{if}\;\;D^{i}\in S_{m}\\ -1 & \text{otherwise} \end{array}(m=1,\;2,\;\cdots ,\;14)$$ (5)

Likewise, for a query drug or compound, we have

$$D^{\text{q}}=\;{\left[\alpha_{1}^{\text{q}}\;\alpha_{2}^{\text{q}}\;\;\;\;\alpha_{3}^{\text{q}}\;\;\;\;\cdots \;\;\;\alpha_{14}^{\text{q}}\right]}^{T}$$ (6)

Its attribution label vector in the ACT system is predicted as

$$L^{\text{q}}={\left[\begin{array}{ccccc} \ell_{1}^{\text{q}} & \ell_{2}^{\text{q}} & \ell_{3}^{\text{q}} & \cdots & \ell_{14}^{\text{q}} \end{array}\right]}^{T}$$ (7)

where

$$\ell_{m}^{q}=\{\begin{array}{ll} +1 & \text{if}\;\;\Delta_{m}\geq 0\;\\ -1 & \text{otherwise} \end{array}(m=1,\;2,\;\cdots ,\;14)$$ (8)

The Δ_*m*_ in **Eq.8** is given by

$$D_{m}=\left[\overset{1144}{\underset{i=1}{\sum }}\ell_{m}^{i}\cdot \exp\left(-\frac{{\left\|D^{\text{q}}-D^{i}\right\|}^{2}}{2\theta^{2}}\right)\right]{\left[\overset{1144}{\underset{i=1}{\sum }}\exp\left(-\frac{{\left\|D^{\text{q}}-D^{i}\right\|}^{2}}{2\theta^{2}}\right)\right]}^{-1}$$ (9)

where θ is a parameter whose optimal value will be determined later, ||**D**^q^ – **D***^i^*||^2^ is the Euclidean distance in the 14-D space (see Eq.2) between the query drug and the *i*-th drug of the benchmark dataset $S^{\text{DO}}$, as given by [28]

$${\left\|D^{\text{q}}-D^{i}\right\|}^{2}=\overset{14}{\underset{u=1}{\sum }}{\left(\alpha_{u}^{\text{q}}-\alpha_{u}^{i}\right)}^{2}$$ (10)

Thus, the attribution label vector L^q^ of Eq.7 for the query drug D^q^ is well defined, and hence its ATC class or classes can be explicitly predicted as well. For example: if $\ell_{1}^{\text{q}}=\ell_{2}^{\text{q}}=\ell_{14}^{\text{q}}=+1$ while all the other components in Eq.7 are equal to −1, this means that the query drug belongs to the 1^st^, 2^nd^, and 14^th^ ATC classes; if $\ell_{3}^{\text{q}}=+1$ while all the others are equal to −1, meaning that the query drug belongs to the 3^rd^ ATC class only; and so forth.

The predictor established via the aforementioned procedures is called iATC-mDO, where “i” means “identify”, “ATC” means “Anatomical Therapeutic Chemical” classification, “m” means “multiple” labels, and “DO” means “drug ontology”.

### Hybridization with iATC-mISF

Question might be raised as asking how to deal with the remaining 2,689 compounds that are not included in the existing ChEBI database? Actually, similar question also existed in using GO (Gene Ontology) to predict the protein subcellular localization [5, 70], enzyme family classes [71, 72], analyzing protein pathway networks [73], and protein-protein interaction [74]. In those cases, the pseudo amino acid composition (PseAAC) approach [24, 25, 75] was applied to deal with those proteins without GO numbers. Likewise, we can also introduce a hybrid predictor for the ATC classification as given by

$$iATC-mHyb\;=\{\begin{array}{ll} iATC-mDO, & \text{for the compounds in ChEBI}\\ iATC-mISF, & \text{Otherwise} \end{array}$$ (11)

where “Hyb” means “hybridization” with the iATC-mISF predictor [4].

### Test procedure

One of the important procedures [66] in developing a new prediction method is how to objectively evaluate its anticipated success rate [66]. To address this, we need to consider two issues. (1) What metrics should be used to quantitatively reflect the predictor's quality? (2) What kind of test approach should be utilized to score the metrics?

#### A set of five metrics for multi-label systems

The metrics used to measure the prediction quality for multi-label systems are much more complicated than those for single-label systems. To make them more intuitive and easier to understand for most experimental scientists, the following five metrics were introduced by Chou [3]: (1) “aiming”, which is for checking the rate or percentage of the correctly predicted labels over the practically predicted labels; (2) “coverage”, for checking the rate of the correctly predicted labels over the actual labels in the system concerned; (3) “accuracy”, for checking the average ratio of correctly predicted labels over the total labels including correctly and incorrectly predicted labels as well as those real labels but are missed in the prediction; (4) “absolute true”, for checking the ratio of the perfectly or completely correct prediction events over the total prediction events; (5) “absolute false”, for checking the ratio of the completely wrong prediction over the total prediction events.

The aforementioned Chou's five metrics can be formulated as [3]

$$\{\begin{array}{l} \text{Aiming}=\;\frac{1}{N}\sum_{k=1}^{N}\left(\frac{\left\|L_{k}\cap L_{k}^{*}\right\|}{\left\|L_{k}^{*}\right\|}\right)\\ \text{Coverage}=\;\frac{1}{N}\sum_{k=1}^{N}\left(\frac{\left\|L_{k}\cap L_{k}^{*}\right\|}{\left\|L_{k}\right\|}\right)\;\\ \text{Accuracy}=\;\frac{1}{N}\sum_{k=1}^{N}\left(\frac{\left\|L_{k}\cap L_{k}^{*}\right\|}{\left\|L_{k}\cup L_{k}^{*}\right\|}\right)\\ \text{Absolute true}=\;\frac{1}{N}\sum_{k=1}^{N}\Delta \left(L_{k},\;L_{k}^{*}\right)\\ \text{Absolute false}=\;\frac{1}{N}\sum_{k=1}^{N}\left(\frac{\left\|L_{k}\cup L_{k}^{*}\right\|-\left\|L_{k}\cap L_{k}^{*}\right\|}{M}\right) \end{array}$$ (12)

Where *N* is the total number of the samples concerned, *M* is the total number of labels for the investigated system, ‖ ‖ means the operator acting on the set therein to count the number of its elements, ∪ means the symbol for the “union” in the set theory, ∩ denotes the symbol for the “intersection”, $L_{k}$ denotes the subset that contains all the labels observed by experiments for the *k*-th sample, $L_{k}^{*}$ represents the subset that contains all the labels predicted for the *k*-th sample, and

$$\begin{array}{l} \sum_{k=1}^{N}D\left(L_{k},\;L_{k}^{*}\right)\\ =\{\begin{array}{cc} 1, & \text{if all the labels in}\;L_{k}^{*}\;\text{are identical to those in}\;L_{k}\\ 0, & \text{otherwise} \end{array} \end{array}$$ (13)

The above approach had been effectively used to study various multi-label systems, such as those in which a protein may occur in two or more different subcellular locations [18–23, 76], or an antimicrobial peptide may have two or more different types [34], or a membrane protein may have two or more different types [77].

#### Test by cross validation

Three cross-validation methods are often used in statistical prediction. They are: (1) independent dataset test, (2) subsampling (or K-fold cross-validation) test, and (3) jackknife test [28]. Of these three, however, the jackknife test is deemed the least arbitrary that can always yield a unique outcome for a given benchmark dataset as elucidated in [66]. Accordingly, the jackknife test has been widely recognized and increasingly used by investigators to examine the quality of various predictors (see, e.g., [11, 65, 78–90]). Accordingly, the jackknife test was also used in this study.

#### Parameter determination

**Figure 3:**
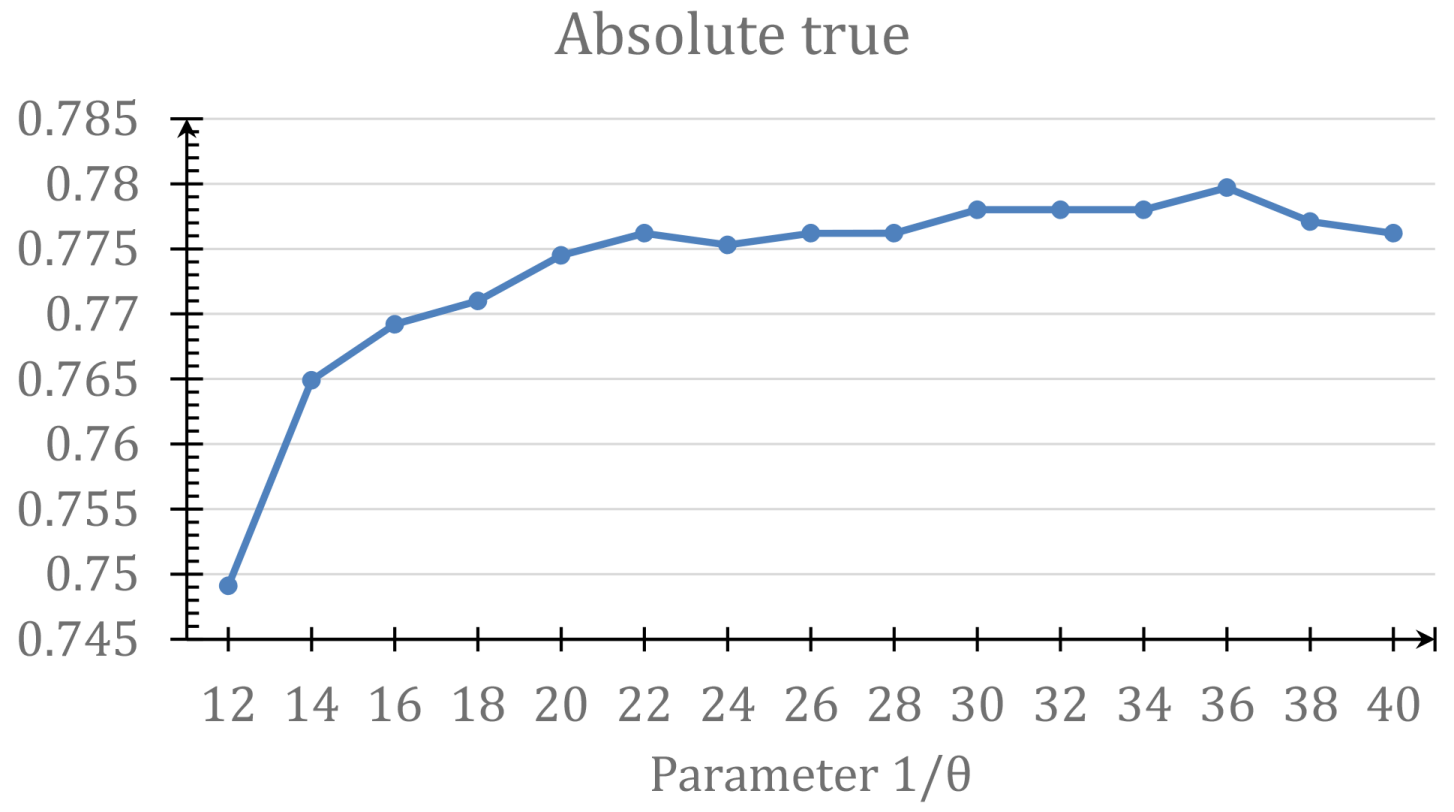
A plot to show the process of finding the optimal θ value in Eq.9 See the main text for further explanation.

Since Eq.9 contains a parameter θ, the predicted results obtained by iATC-mDO will depend on the parameter's value. In this study, the optimal value for θ was determined by maximizing the absolute true rate (see the 4^th^ sub-equation in Eq.12) by the jackknife validation on the benchmark dataset $S^{\text{DO}}$. As shown in Figure 3, when θ = 1/36, the absolute true rate reached its highest score. And such a value would be used for iATC-mDO predictor in further study.

## CONCLUSION

A new method for predicting the ATC classes has been developed by hybridizing the drug ontology approach with the best existing ATC predictor. The new predictor has outperformed the best existing ATC predictor in all the five metrics used to examine the prediction quality of a predictor for multi-label systems, particularly in the “absolute true” rate and the “absolute false” rate, the two most difficult-to-improve indexes. To maximize the users’ convenience, a publically accessible web-server has been established at http://www.jci-bioinfo.cn/iATC-mHyb along with a step-by-step guide. Moreover, the MATLAB code for the new method is also available as in Supporting Information S4, which can be directly downloaded from the web-server.
